# Tarlatamab in Small Cell Lung Cancer and Extrapulmonary Small Cell Carcinoma: A Single-Centre Retrospective Cohort Study of Routine-Practice Outcomes

**DOI:** 10.3390/cancers18193078

**Published:** 2026-09-22

**Authors:** Ayrton Bangolo, Lili Zhang, Jiahe Zhao, Behzad Amoozgar, Sarvarinder Gill, Shibhani Rajanna, Shareif Abdelwahab, Andres Molina, Tanner Robinson, Umangpreet Gill, Andrea Torres, Akhil Akula, Mahe Tahniyat, Maamoun Seklawi, Tri Vo, Thilini Suriyaarachchi, Oriana Castillo-Briceno, Sujeewa Kalubowila, Tori Salorenzo, Darya Klechykava, Avery Kellman, Kaushik Roy, Victor H. Villarreal, Alia Ramzan, Hamza Haq, Rajendra Gunti, Soroush Abaresh, Gurmanjot Kaur, Jonathan Montalvo, Zhongxuan-Jonathan Wang, Aanya Srivastava, Lipi Gudiboina, Benjamin S. Feuer, Vennela Talla, Rajin Persaud, Winnie Noe, Shalini Subramanian, Simcha Weissman, Martin Gutierrez, Miguel Gonzalez-Velez

**Affiliations:** 1 Department of Hematology and Oncology, Hackensack Meridian John Theurer Cancer Center, Hackensack, NJ 07047, USA; 2 Department of Bone Marrow Transplant and Cellular Therapy, Loma Linda University Health, Loma Linda, CA 92350, USA; 3 Division of Leukemia, Hackensack Meridian John Theurer Cancer Center, Hackensack, NJ 07047, USA; 4 Department of Internal Medicine, Hackensack Meridian Health, Palisades Medical Center, North Bergen, NJ 07047, USA; 5 Department of Radiation Oncology, Cleveland Clinic Foundation, Cleveland, OH 44195, USA; 6 Division of Thoracic and GI Oncology and Phase 1 Clinical Trial, Hackensack Meridian John Theurer Cancer Center, Hackensack, NJ 07047, USA

**Keywords:** tarlatamab, small cell lung cancer, extrapulmonary small cell carcinoma, DLL3, bispecific T-cell engager, BiTE, real-world data, cytokine release syndrome

## Abstract

Background: Tarlatamab, a Delta-like ligand 3 (DLL3)-targeting bispecific T-cell engager (BiTE), has demonstrated significant efficacy in relapsed small cell lung cancer (SCLC) in clinical trials. However, real-world clinical outcomes and its efficacy in extrapulmonary small cell carcinoma (EPSCC) remain sparsely characterized. Methods: We conducted a retrospective, single-institution cohort study of 24 adult patients with moderately pretreated advanced SCLC (n = 19) or EPSCC (n = 5) who received standard-of-care tarlatamab. Endpoints included objective response rate (ORR), progression-free survival (PFS), overall survival (OS), and safety. Results: Patients had received a median of two prior lines of therapy, and 38% had baseline brain metastases. The overall intent-to-treat (ITT) ORR was 25.0%. Among response-evaluable patients (n = 18), the ORR was 33.3%. Efficacy was notably discordant between histologies: the SCLC cohort achieved an ITT ORR of 31.6% (46.2% in evaluable patients), whereas 100% of EPSCC patients experienced progressive disease. Systemic responses were observed in patients with baseline brain metastases (ORR 33.3%). The safety profile was manageable; any-grade CRS and ICANS occurred in 58% and 29% of patients, respectively. Severe (Grade ≥ 3) toxicities were rare, and tocilizumab was utilized therapeutically in 50% of patients for low-grade toxicity. Conclusions: These real-world data are consistent with tarlatamab’s systemic efficacy and tolerability in SCLC. The lack of clinical benefit observed in this small EPSCC cohort raises a hypothesis that requires prospective biomarker-correlated evaluation.

## Simple Summary

Small cell lung cancer (SCLC) is an aggressive cancer with few options once it returns after initial therapy. Tarlatamab is a recently approved immunotherapy that links a patient’s own T cells to cancer cells expressing a protein called DLL3. It is effective in clinical trials, but less is known about how it performs in everyday practice, and it has not been studied in a related rare cancer that begins outside the lungs, extrapulmonary small cell carcinoma (EPSCC). We reviewed 24 patients treated at a single centre. About one-third of patients with SCLC had tumour shrinkage, including some with brain metastases, and side effects were manageable and mostly mild. None of the five patients with EPSCC had tumour shrinkage; however, five patients with tumours arising from different organs are far too few to draw conclusions, no DLL3 testing was performed, and the time without disease worsening was very short. Our findings add to the limited routine-practice information on tarlatamab in SCLC and raise a question, not an answer, about its use outside the lung, which should be tested in prospective studies that measure DLL3.

## 1. Introduction

Small cell lung cancer (SCLC) is a highly aggressive, poorly differentiated neuroendocrine carcinoma that constitutes approximately 13–15% of all lung malignancies. It is clinically defined by its rapid tumor growth, high propensity for early and widespread metastatic dissemination, and the frequent development of paraneoplastic syndromes. Although SCLC is highly sensitive to initial cytotoxic therapy, the standard first-line treatment of platinum-etoposide chemotherapy—even with the recent integration of immune checkpoint inhibitors—is rarely curative [1]. The vast majority of patients with extensive-stage disease experience rapid relapse, typically within months of completing initial therapy. Historically, subsequent therapeutic options, such as topotecan or lurbinectedin, have offered only modest clinical benefits, characterized by low objective response rates, substantial hematologic toxicities, and short durations of response. The historically dismal prognosis for relapsed SCLC has underscored a critical, decades-long unmet need for novel, targeted, and highly effective therapeutic modalities [2].

This therapeutic challenge extends to extrapulmonary small cell carcinoma (EPSCC), a rare, heterogeneous, and highly aggressive group of tumors that share striking histological and molecular hallmarks with SCLC—including ubiquitous TP53 and RB1 inactivation—but arise from tissues outside the pulmonary tree [3]. EPSCC can originate in virtually any organ, though it is most frequently identified in the genitourinary tract (e.g., prostate, bladder), the gastrointestinal system (e.g., esophagus, pancreas, gastroesophageal junction), and the gynecologic tract. Because EPSCC accounts for only 2.5% to 5% of all small cell carcinomas, dedicated prospective clinical trials are exceedingly scarce. Consequently, treatment paradigms are almost entirely extrapolated from SCLC guidelines. Despite aggressive initial multimodal therapy, the prognosis for metastatic or relapsed EPSCC remains exceptionally poor, necessitating the urgent exploration of biomarker-driven therapeutic avenues that target the underlying neuroendocrine biology rather than the anatomical site of origin [3,4].

A highly promising and unifying target across these neuroendocrine malignancies is Delta-like ligand 3 (DLL3), an inhibitory ligand of the Notch signaling pathway. In healthy adult tissues, DLL3 expression is strictly confined to the intracellular Golgi apparatus and is notably absent from the cell surface, preventing unwanted targeting [5]. However, driven by the lineage-specific transcription factor ASCL1, DLL3 is aberrantly trafficked to and overexpressed on the cell surface of more than 80% of SCLCs and other high-grade neuroendocrine carcinomas. This uniquely differential expression pattern establishes DLL3 as a highly specific tumor-associated antigen, providing an exceptionally wide therapeutic window for targeted therapies with minimal risk of on-target, off-tumor toxicity [5,6].

Tarlatamab is a first-in-class, half-life extended Bispecific T-cell Engager (BiTE^®^) uniquely engineered to exploit this therapeutic vulnerability. The molecule possesses two distinct binding domains: one that specifically recognizes cell-surface DLL3 on the tumor, and another that binds to the CD3 epsilon subunit of the T-cell receptor complex [7]. By physically bridging the cancer cell and the patient’s endogenous T-cells, tarlatamab induces the formation of a cytolytic synapse. This interaction triggers robust, polyclonal T-cell activation and the subsequent release of perforin and granzymes, leading to potent T-cell-mediated tumor lysis [8]. Crucially, this mechanism bypasses the need for major histocompatibility complex (MHC) class I antigen presentation—a pathway frequently downregulated in SCLC as an immune escape mechanism—thereby effectively turning a traditionally immune-evasive, “cold” tumor into a target for cellular immunity.

The clinical efficacy of this innovative approach was firmly established in the pivotal, open-label, phase 2 DeLLphi-301 study. In a cohort of patients with relapsed or refractory SCLC who had progressed after at least two prior lines of therapy, tarlatamab (at the optimized 10 mg dose) demonstrated a robust objective response rate (ORR) of 40% and a median overall survival (OS) of 14.3 months. Based on these compelling and unprecedented results in the refractory setting, tarlatamab received accelerated approval from the U.S. Food and Drug Administration in May 2024, heralding a new era in the treatment of SCLC [9].

However, while clinical trials provide crucial efficacy and safety data in a highly controlled environment, there is a pressing need to investigate the effectiveness of tarlatamab in a real-world setting. Furthermore, a significant knowledge gap remains regarding the utility of tarlatamab in patients with EPSCC, a population excluded from the DeLLphi-301 trial [9]. While EPSCC shares the biological rationale for DLL3 targeting, its distinct tumor microenvironments and varied anatomical origins may significantly impact the efficacy of immune-engaging therapies. This study aims to address these gaps by reporting the real-world efficacy, safety outcomes, and histological divergences in a cohort of moderately pretreated patients with both SCLC and EPSCC treated with tarlatamab.

## 2. Methods

### 2.1. Study Design and Patient Selection

We conducted a retrospective, single institutional observational cohort study evaluating adult patients (≥18 years of age) treated with tarlatamab at the John Theurer Cancer Center. Patients were required to have a histologically or cytologically confirmed diagnosis of advanced SCLC or EPSCC and must have initiated at least one dose of tarlatamab between 16 May 2024, and 30 August 2025. The data cutoff for all survival and response analyses was 5 December 2025, allowing for a longer period of follow-up. Patients enrolled in prospective clinical trials for tarlatamab during this timeframe were excluded to strictly isolate real-world outcomes. The study was approved by the Hackensack Meridian Health IRB (Pro2025-0529) on 1 December 2025, with waiver of informed consent and waiver of HIPAA authorisation. All clinical information analysed was generated during routine care; research-specific chart review, data abstraction, de-identification and analysis were initiated only after IRB approval.

### 2.2. Treatment Protocols

Tarlatamab was given intravenously per the Food and Drug Administration (FDA) approved step-up schedule: 1 mg on cycle 1 day 1, 10 mg on cycle 1 day 8 and day 15, then 10 mg every 2 weeks in continuous 28-day cycles until progression, unacceptable toxicity, withdrawal or death. Premedication and monitoring followed institutional standards. No predefined prophylactic or pre-emptive tocilizumab protocol was in place; tocilizumab and corticosteroids were given therapeutically at the discretion of the treating physician. Patients were closely monitored for immune-related adverse events during the initial step-up phase. The management of treatment-related toxicities, including dose delays, the administration of tocilizumab, and the use of high-dose corticosteroids for immune effector cell-associated neurotoxicity syndrome (ICANS) or cytokine release syndrome (CRS), was carried out at the discretion of the treating physician based on established institutional and societal guidelines.

### 2.3. Data Collection and Statistical Analysis

Comprehensive clinical data were extracted from electronic medical records (EMR) using a standardized data collection template. Baseline variables collected included patient demographics (age, sex, race), primary tumor site (pulmonary vs. extrapulmonary), number and types of prior lines of systemic therapy, Eastern Cooperative Oncology Group (ECOG) performance status at the initiation of tarlatamab, baseline laboratory values, and the presence of brain metastases. Treatment-specific data included the total number of administered tarlatamab cycles, dose interruptions or reductions, and the requirement for mitigating agents such as tocilizumab or systemic steroids.

The primary efficacy endpoints were Overall Response Rate (ORR), Progression-Free Survival (PFS), and Overall Survival (OS). Tumor responses were determined by retrospective investigator review of radiology reports and images applying RECIST 1.1 principles; measurements were those recorded by the reporting radiologist and treating physician and were not re-measured by independent central review; imaging intervals were clinically determined (approximately every 6–9 weeks, but not standardised); and clinical progression without radiographic confirmation was permitted as a PFS event. PFS was defined as the time from the first dose of tarlatamab to radiologic or clinical disease progression or death from any cause. OS was defined as the time from the first dose of tarlatamab to death from any cause. The secondary safety endpoints focused on the incidence and maximum severity of adverse events. Specifically, CRS and ICANS were graded retrospectively utilizing the American Society for Transplantation and Cellular Therapy (ASTCT) consensus grading criteria. 6 patients who were non-evaluable (NE) for response: these patients experienced rapid clinical decline (n = 4), death (n = 1), or were lost to follow-up (n = 1) prior to their first protocol-defined radiographic assessment. Patients without post-baseline imaging were classified as NE and explicitly retained in the denominator for all intention-to-treat (ITT) analyses to avoid overestimating the response rate.

Statistical analyses were performed using standard descriptive and inferential methods. Continuous variables were summarized using medians and ranges, while categorical variables were reported as frequencies and percentages. Survival functions for PFS and OS were estimated using the Kaplan–Meier method, with corresponding 95% confidence intervals (CIs). The data cutoff for all analyses was 5 December, 2025. All analyses were conducted using SPSS version 27.0 (IBM Corp., Armonk, NY, USA).

## 3. Results

### 3.1. Patient Characteristics and Treatment Exposure

A total of 24 patients who received at least one dose of tarlatamab were included in this retrospective analysis. The baseline demographic and clinical characteristics of the cohort are comprehensively detailed in Table 1. The median age of the patients at the initiation of tarlatamab was 67 years (range, 55–85 years), and the cohort was predominantly female (n = 14, 58%). The majority of patients (n = 19, 79%) had a primary diagnosis of small cell lung cancer (SCLC). The remaining 5 patients (21%) were diagnosed with extrapulmonary small cell carcinoma (EPSCC), representing a rare and historically understudied subgroup, with primary tumor sites originating in the prostate, uterine cervix, and gastroesophageal junction (GEJ).

This represented a moderately pretreated and high-risk population. Patients had received a median of 2 prior lines of systemic therapy (range, 1–3) before commencing tarlatamab. Nearly all patients received frontline platinum-etoposide (frequently combined with a PD-L1 inhibitor such as atezolizumab or durvalumab), followed by standard second-line agents (such as topotecan or lurbinectedin) upon progression. All patients had experienced documented disease progression on these prior regimens before initiating tarlatamab. Furthermore, the disease burden at the time of treatment initiation was substantial, with one-third of the cohort (n = 9, 38%) having a history of known brain metastases. Despite this advanced disease state, the majority of the cohort maintained a robust functional status, with 82% exhibiting an Eastern Cooperative Oncology Group (ECOG) performance status of 0 or 1. Regarding treatment exposure, patients received a median of 5 doses of tarlatamab (range, 1–14), reflecting sustained tolerability and clinical benefit in a subset of the population.

### 3.2. Efficacy

At the data cutoff of 5 December 2025, there were 18 PFS events and 12 OS events in the overall cohort, with a median follow-up among surviving patients of 7.5 months. For the overall cohort (N = 24), the ITT ORR was 25.0% (95% CI, 9.8–46.7%). When excluding the 6 patients classified as NE, the ORR in the response-evaluable population (n = 18) was 33.3% (95% CI, 13.3–59.0%). The overall ITT DCR was 29.2% (95% CI, 12.6–51.1%), and the evaluable DCR was 38.9% (95% CI, 17.3–64.3%). Among the 19 patients with SCLC, the ITT ORR was 31.6% (95% CI, 12.6–56.6%) and the ITT DCR was 36.8% (95% CI, 16.3–61.6%). Thirteen SCLC patients were fully evaluable for response, yielding an evaluable SCLC ORR of 46.2% (95% CI, 19.2–74.9%) and an evaluable DCR of 53.8% (95% CI, 25.1–80.8%). Six SCLC patients (32%) were Not Evaluable (NE) primarily due to rapid clinical decline or loss to follow-up prior to their first imaging scan. Conversely, no clinical benefit was observed in the EPSCC subgroup (n = 5), where the ITT and evaluable ORR was 0%. DLL3 expression status was not routinely assessed by pathology for these EPSCC patients. Patient-level characteristics for the EPSCC cohort are detailed in Table 2.

Notably, tarlatamab demonstrated clinical activity in patients with central nervous system (CNS) involvement. Among the 9 patients with baseline brain metastases, the ORR was 33.3% (3 PRs), all of which occurred in SCLC patients.

The median progression-free survival (PFS) from the initiation of tarlatamab was 3.0 months (95% confidence interval [CI], 2.0 to 4.0 months), as depicted in the Kaplan–Meier curve in Figure 1, with numbers at risk reported in months in Table 3. Figure 2 depicts the PFS of SCLC and EPSCC, with numbers at risk reported in months in Table 4. Importantly, at the time of this analysis, the median overall survival (OS) calculated from the start of tarlatamab therapy had not yet been reached (Figure 3), with numbers at risk reported in months in Table 5. The estimated 6-month OS rate was 55% (Figure 3).

### 3.3. Safety and Tolerability

The safety profile of tarlatamab in this real-world, medically diverse cohort was thoroughly evaluated and found to be consistent with the known mechanism of action of T-cell engaging therapies and previously published pivotal trial data. The most common and clinically notable adverse events were immune-mediated, specifically Cytokine Release Syndrome (CRS) and Immune effector Cell-Associated Neurotoxicity Syndrome (ICANS).

CRS of any grade was observed in over half of the cohort (n = 14, 58%). The vast majority of these events were mild to moderate (Grade 1 or 2) and occurred during the initial step-up dosing phase. Grade ≥ 3 CRS was rare, occurring in only 2 patients (8%); these severe cases manifested as clinically significant hypotension requiring transient vasopressor support.

ICANS of any grade was reported in 7 patients (29%). Similar to CRS, these neurotoxic events were predominantly low-grade and presented with symptoms such as confusion, lethargy, or expressive difficulties, including impaired handwriting. Only 1 patient (4%) experienced a severe, Grade ≥ 3 ICANS event, which presented acutely with global aphasia and obtundation. Notably, this neurologic toxicity was completely reversible following prompt medical intervention.

The management of these immune-mediated toxicities by treating physicians aligned with established societal guidelines. Tocilizumab, an interleukin-6 (IL-6) receptor antagonist, was administered to 12 patients (50%) for the management of CRS and/or ICANS, frequently in conjunction with high-dose systemic corticosteroids. Systemic corticosteroids were utilized for toxicity management in 11 of the 19 (58%) SCLC patients, frequently for the management of ICANS or CRS that had not fully resolved following initial intervention. Overall, the therapy was manageable; only 2 patients (8%) required permanent discontinuation of tarlatamab due to recurrent or severe treatment-related toxicity. Crucially, there were no Grade 5 adverse events, with zero treatment-related deaths occurring in this cohort.

## 4. Discussion

This study provides critical real-world evidence regarding the clinical utility of tarlatamab in an inherently high-risk population of patients with advanced small cell carcinomas. Our principal finding is that tarlatamab demonstrates meaningful anti-tumor activity and a highly manageable safety profile in routine clinical practice. These observations are broadly concordant with the phase 2 DeLLphi-301 trial, although a single-centre retrospective series of 24 patients cannot validate a prospective trial [9].

The efficacy observed in our cohort—specifically, an ORR of 25% in the entire cohort but 32% for SCLC and a median PFS of 3.0 months—is clinically significant for a refractory population that has exhausted standard therapeutic options. Historically, outcomes in relapsed SCLC have been dismal; standard-of-care agents such as topotecan and lurbinectedin typically yield real-world ORRs of 15–20% and median PFS durations of only 2.0 to 3.5 months, often accompanied by severe myelosuppression [10,11]. While our observed ORR of 25% (32% for SCLC) appears lower than the 40% reported in the strictly selected DeLLphi-301 trial, this variance is entirely consistent with the well-documented efficacy gap between clinical trials and real-world oncology practice [12]. Our cohort included patients with poorer prognostic features, higher disease burdens, and significant comorbidities that would have precluded clinical trial enrollment. Furthermore, a highly novel, yet cautionary, contribution of our study is the inclusion of patients with EPSCC. Because EPSCC accounts for only 2.5% to 5% of all small cell carcinomas, its treatment paradigms are almost entirely extrapolated from SCLC data. No responses were observed among the five patients with EPSCC. DLL3 was not assessed in any patient, and progression-free survival did not differ significantly between the groups; these findings are therefore hypothesis-generating and cannot be attributed to differential DLL3 biology. While the SCLC cohort achieved an ORR of 32%, the EPSCC cohort (n = 5) experienced uniform treatment failure, with 100% of patients exhibiting progressive disease as their best response.

Several factors likely contribute to this complete lack of efficacy in our EPSCC cohort. First, while DLL3 expression is nearly ubiquitous in SCLC, translational data indicate that DLL3 expression in EPSCC—such as neuroendocrine prostate or cervical cancers—can be highly heterogeneous and of lower surface density, potentially impairing the formation of the cytolytic synapse required by tarlatamab [13,14]. Second, extrapulmonary primary sites often harbor inherently different and highly immunosuppressive tumor microenvironments (TMEs) compared to the lungs, which may inhibit endogenous T-cell infiltration and activation. Finally, the EPSCC patients in our cohort were moderately pretreated, with many having failed prior aggressive immuno-oncology regimens, reflecting a highly resistant clonal population. Although our small sample size (n = 5) precludes definitive statistical conclusions, these real-world suggests caution and supports prospective evaluation of SCLC-approved T-cell engagers to EPSCC. They support prospective, biomarker-correlated basket studies incorporating DLL3 assessment before tarlatamab is used in extrapulmonary disease.

A critical nuance of our real-world cohort is the high prevalence of central nervous system (CNS) involvement. At the initiation of tarlatamab, 38% (9 of 24) of our patients had known brain metastases, a higher proportion than the approximately 22–26% typically permitted in pivotal trials like DeLLphi-301 [9]. The management of brain metastases in SCLC remains a formidable clinical challenge, as the blood–brain barrier (BBB) severely limits the CNS penetrance of large molecular therapies, including BiTEs (~55–100 kDa) [15]. However, while the tarlatamab molecule itself may exhibit limited BBB crossing, activated endogenous T-cells are known to actively traffic into the CNS, potentially mediating an intracranial anti-tumor immune response [16]. Exploratory subsets from clinical trials have suggested that tarlatamab possesses some intracranial activity in patients with treated, stable brain lesions. In our real-world setting, patients presented with heterogeneous baseline CNS disease, including a mix of previously treated, stable, and active untreated brain metastases. Three of nine patients with baseline brain metastases had a systemic partial response. Because intracranial lesions were not independently measured, these data do not establish intracranial activity. Whether activated T cells traffic into the CNS, as has been described in other settings [16], remains a hypothesis that requires studies with protocol-specified brain imaging and intracranial response endpoints [17]. Moving forward, dedicated real-world studies assessing specific intracranial response rates (iORR) will be vital to fully elucidating the role of tarlatamab in managing SCLC brain metastases [17].

The safety profile of tarlatamab in our study aligns closely with established trial data, suggesting that its unique toxicities can be managed outside of clinical trials. The incidence of any-grade CRS (58% in our cohort vs. 51–55% in DeLLphi-301) and ICANS (29% vs. 47%) were highly comparable [9]. Grade 3 or more events were uncommon in this cohort. This uncontrolled retrospective design cannot establish that any particular monitoring or intervention strategy caused this safety profile. Standardized ASTCT grading and early intervention algorithms should be followed [18,19]. Notably, 50% of our patients received tocilizumab. This high utilization rate reflects a low threshold for IL-6 blockade in current practice [20,21,22]; whether early tocilizumab affects efficacy could not be assessed here.

Several biological mechanisms could underlie a differential effect of DLL3-directed T-cell engagers by primary site, none of which we were able to test. First, cell-surface DLL3 is expressed in the majority of SCLC but appears more variable in extrapulmonary high-grade neuroendocrine carcinomas [23,24,25]; low or heterogeneous surface density could impair cytolytic synapse formation. Second, extrapulmonary primaries may differ in T-cell infiltration and immunosuppressive microenvironment [26,27]. Third, loss of interferon signaling or Beta-2-microglobulin (B2M) could impair T-cell effector function, although—because bispecific T-cell engagers act independently of peptide–MHC presentation—MHC class I downregulation is not expected to be a primary resistance mechanism for tarlatamab [28,29,30]. Because no DLL3, MHC class I, programmed death-ligand 1 (PD-L1) or neuroendocrine-subtype immunohistochemistry was available and no paired on-treatment biopsies were obtained, these remain untested hypotheses. Similarly, neuron-specific enolase and pro-gastrin-releasing peptide are not measured routinely at our center and could not be correlated with outcome [31,32].

This study has some limitations. It is retrospective, single-center and uncontrolled, with attendant selection bias and limited generalizability. Six of 24 patients were not evaluable for response, and patients treated near the end of the accrual period had short follow-up, so both response and time-to-event estimates are immature. Imaging intervals were clinically determined rather than standardised; responses were investigator-assessed without independent central radiological review; and clinical progression was accepted as a PFS event. Brain lesions were not measured independently, so no intracranial response rate can be derived. The EPSCC group comprises only five patients with tumours of three different primary organs and cannot be treated as a homogeneous biological cohort or as evidence of a site-specific lack of efficacy. No DLL3 biomarker data were available for any patient. CRS and ICANS were graded retrospectively from clinical documentation, which may under-ascertain low-grade events, and details of tocilizumab dosing, corticosteroid indication and critical-care utilisation were incompletely recorded. Finally, there is no comparator arm, so no conclusions about comparative effectiveness can be drawn.

## 5. Conclusions

In conclusion, our real-world experience provides supportive evidence that tarlatamab is an active and tolerable therapeutic agent for heavily pretreated patients with advanced SCLC. The findings are consistent with the utility of tarlatamab in SCLC. However, our study suggests that caution is warranted when considering the off-label extrapolation of SCLC therapies to EPSCC based solely on shared histology. The absence of responses in this small, biologically heterogeneous series without DLL3 testing raises a hypothesis that requires prospective biomarker-correlated evaluation and does not permit definitive conclusions regarding efficacy in EPSCC.

## Figures and Tables

**Figure 1:**
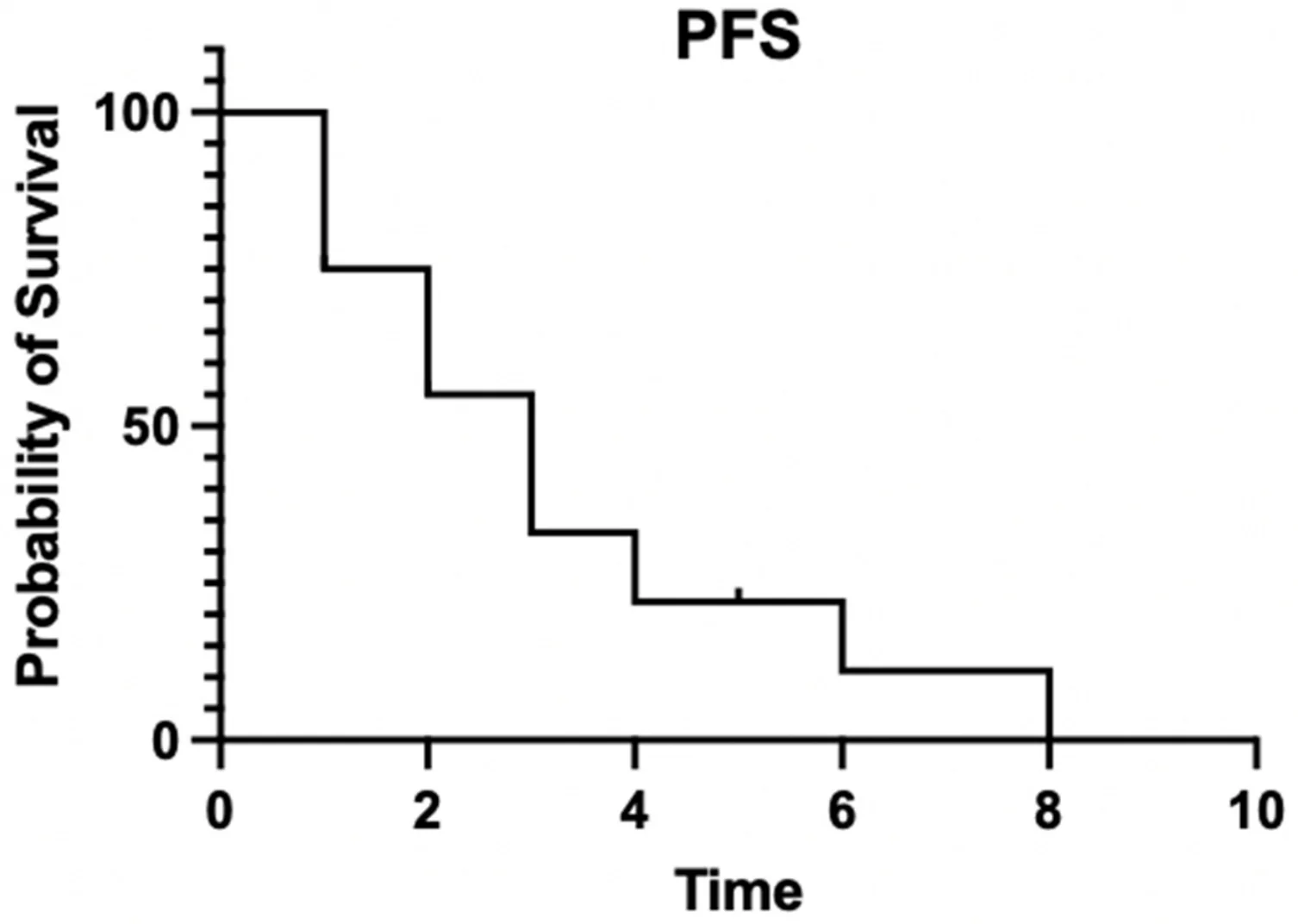
Kaplan–Meier estimate of Progression-Free Survival (PFS) from the initiation of tarlatamab. The curve demonstrates PFS for the entire cohort (N = 24) following the first dose of tarlatamab. The median PFS is 3.0 months (95% CI, 2.0–4.0 months).

**Figure 2:**
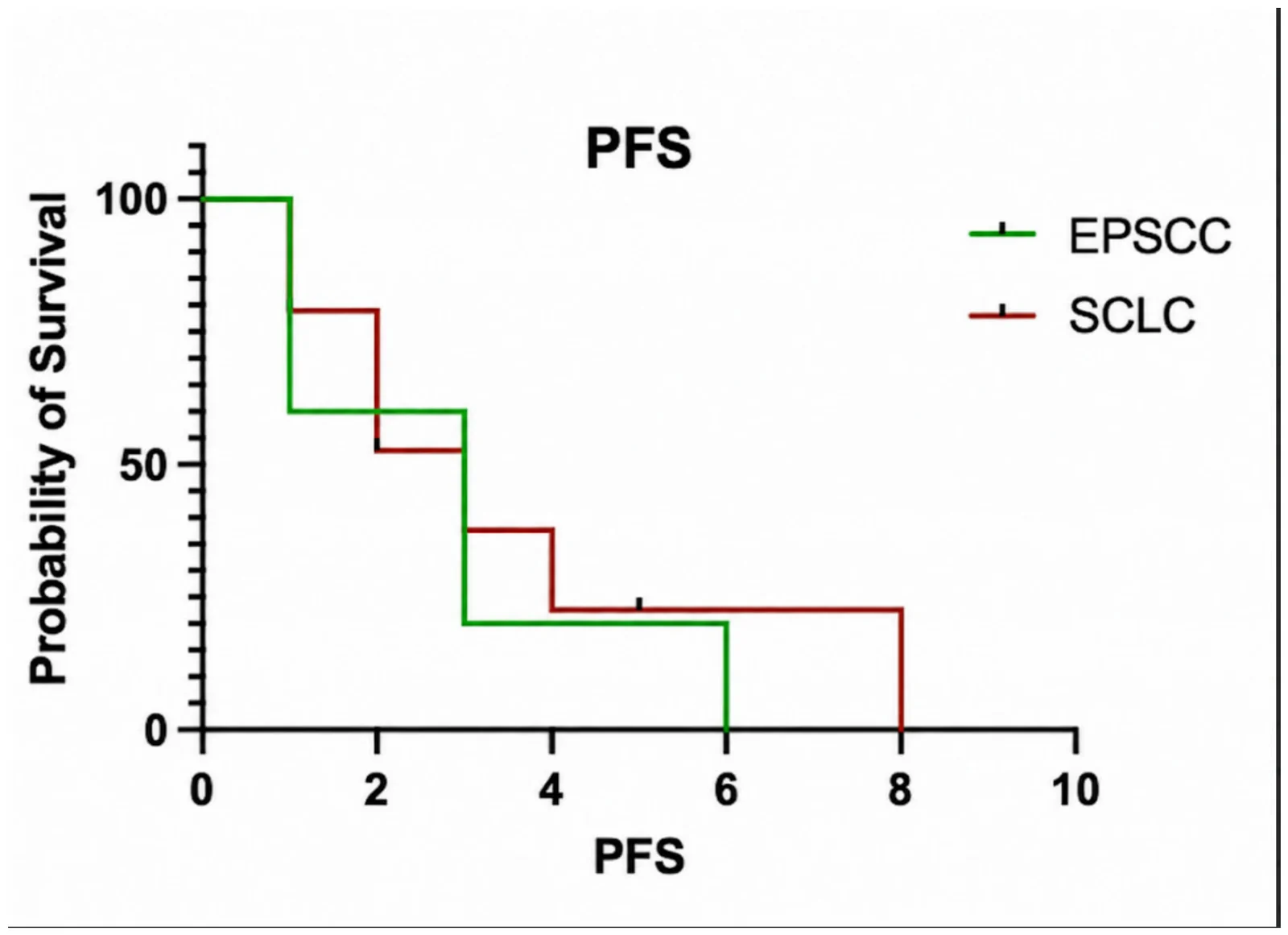
Exploratory Kaplan–Meier estimate of progression-free survival by histology. Median PFS 3.0 months in both groups. EPSCC (n = 5): Median PFS: 3.0, (95% CI: 1.0 to 6.0). SCLC (n = 19): Median PFS: 3.0, (95% CI: 2.0 to 4.0).

**Figure 3:**
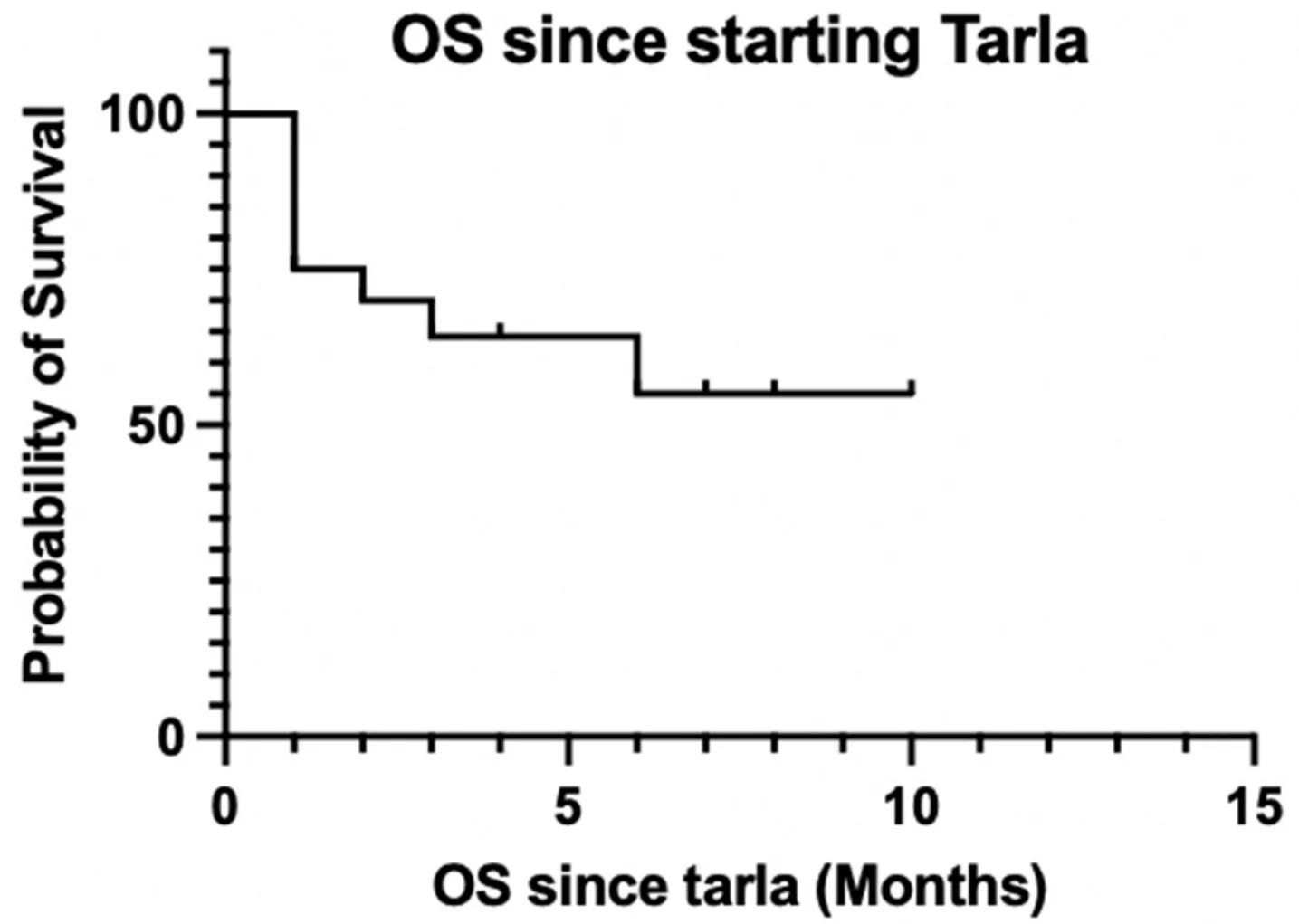
Kaplan–Meier estimate of overall survival from the first tarlatamab dose (N = 24; 12 deaths, 12 censored). Median OS not reached; estimated 6-month OS 55%. Median OS was not reached (95% CI, 6.0 months–NE).

**Table 1:** Baseline Characteristics and Key Treatment Outcomes.

Characteristic	All Patients (N = 24)	SCLC (N = 19)	EPSCC (N = 5)
**Median age at tarlatamab start, years (range)**	67 (55–85)	69 (58–85)	65 (55–71)
**Female, n (%)**	14 (58)	12 (63)	2 (40)
**Race, n (%)**			
White	22 (92)	17 (89)	5 (100)
Other/Unknown	2 (8)	2 (11)	0 (0)
**ECOG PS at Tarlatamab Start, n (%)** *			
0–1	18 (82)	14 (82)	4 (80)
≥2	4 (18)	3 (18)	1 (20)
**Brain Metastases at Tarlatamab Start, n (%)**	9 (38)	6 (32)	3 (60)
**Prior Systemic Therapy (Specific to High grade Neuro-endocrine tumor)**			
Median prior lines, n (range) †	2 (1–3)	2 (1–3)	2 (1–2)
Platinum-Etoposide First-Line, n (%) †	21/23 (91)	17/18 (94)	5/5 (100)
**Efficacy and Response Rates, n (%)**			
Objective Response Rate (ORR)	6 (25)	6 (32)	0 (0)
Best Overall Response			
Complete Response (CR)	0 (0)	0 (0)	0 (0)
Partial Response (PR)	6 (25)	6 (32)	0 (0)
Stable Disease (SD)	1 (4)	1 (5)	0 (0)
Progressive Disease (PD)	11 (46)	6 (32)	5 (100)
Not Evaluable (NE)	6 (25)	6 (32)	0 (0)
Disease Control Rate (DCR) ‡	7 (29)	7 (37)	0 (0)
**Response Stratified by CNS Involvement, n (%)**			
**Patients WITH Brain Metastases (N = 9)**			
Objective Response Rate (ORR)	3 (33)	-	-
Disease Control Rate (DCR)	3 (33)	-	-
Best Response: PR/SD/PD/NE	3/0/4/2	-	-
**Patients WITHOUT Brain Mets (N = 15)**			
Objective Response Rate (ORR)	3 (20)	-	-
Disease Control Rate (DCR)	4 (27)	-	-
Best Response: PR/SD/PD/NE	3/1/7/4	-	-
**Tarlatamab-Related Toxicities, n (%)**			
CRS (Any Grade)	14 (58)	10 (53)	4 (80)
Grade ≥ 3	2 (8)	1 (5)	1 (20)
ICANS (Any Grade)	7 (29)	5 (26)	2 (40)
Grade ≥ 3	1 (4)	1 (5)	0 (0)
Tocilizumab Use for Toxicity	12 (50)	9 (47)	3 (60)
**Efficacy and Response Rates, n (%) [95% CI]**			
Objective Response Rate (ITT ORR)	6 (25.0%) [9.8–46.7%]	6 (31.6%) [12.6–56.6%]	0 (0%) [0.0–52.2%]
Disease Control Rate (ITT DCR)	7 (29.2%) [12.6–51.1%]	7 (36.8%) [16.3–61.6%]	0 (0%) [0.0–52.2%]
**Evaluable Efficacy (Excluding NE)**	(N = 18)	(N = 13)	(N = 5)
Evaluable ORR	6 (33.3%) [13.3–59.0%]	6 (46.2%) [19.2–74.9%	0 (0%) [0.0–52.2%]
Evaluable DCR	7 (38.9%) [17.3–64.3%]	7 (53.8%) [25.1–80.8%]	0 (0%) [0.0–52.2%]

**Abbreviations:** SCLC, small cell lung cancer; EPSCC, extrapulmonary small cell carcinoma; ECOG PS, Eastern Cooperative Oncology Group Performance Status; ORR, Objective Response Rate; DCR, Disease Control Rate; CNS, Central Nervous System; CRS, Cytokine Release Syndrome; ICANS, Immune effector Cell-Associated Neurotoxicity Syndrome; NE, Not Evaluable. Data are n (%) unless otherwise specified. Percentages may not sum to exactly 100 due to rounding. * Percentages for ECOG PS are calculated based on patients with available data (N = 22 for All Patients; N = 17 for SCLC; N = 5 for EPSCC). † Data for prior lines of therapy were available for 23 patients. ‡ Disease Control Rate is defined as the sum of Complete Responses, Partial Responses, and Stable Disease. Dashes (-) indicate sections where subdivision by histology was not performed to prevent over-fragmentation of small subgroups.

**Table 2:** Characteristics and Outcomes of the EPSCC Cohort (n = 5).

Patient	Age/Sex	Primary Site	Pathological Differentiation	Molecular Characteristics	Stage	Prior Systemic Therapies	Best Response	DLL3 Expression	Brain Metastases	ECOG	Tarlatamab Cycles	Time to Progression	Survival Status/Follow-Up
1	67/M	Prostate	Poorly differentiated	Not Available	IV	Platinum/Etoposide, Enzalutamide	PD	Not tested	Yes	1	1 cycle	1.0 month	Deceased (1.5 months)
2	55/F	Uterine Cervix	Poorly differentiated	Not Available	IV	Platinum/Etoposide, Pembrolizumab, Paclitaxel	PD	Not tested	No	1	2 cycles	2.0 months	Deceased (2.8 months)
3	71/M	GEJ	Poorly differentiated	Not Available	IV	Platinum/Etoposide, Nivolumab, Lurbinectedin	PD	Not tested	Yes	3	3 cycles	3.0 months	Deceased (4.2 months)
4	65/M	Prostate	Poorly differentiated	Not Available	IV	Platinum/Etoposide, Docetaxel	PD	Not tested	Yes	1	4 cycles	4.0 months	Alive (5.5 months)
5	60/F	Uterine Cervix	Poorly differentiated	Not Available	IV	Platinum/Etoposide, Topotecan	PD	Not tested	No	1	6 cycles	6.0 months	Deceased (7.1 months)

(Note: GEJ = Gastroesophageal junction; PD = Progressive Disease. The median Time to Progression (PFS) for this cohort is 3.0 months. All patients ultimately experienced clinical or radiographic progressive disease as their best overall response).

**Table 3:** Numbers At Risk (Months) for PFS.

0	24
1	24
2	15
3	10
4	6
5	4
6	2
8	1

**Table 4:** Numbers at risk (months) for PFS of EPSCC and SCLC.

0	5	19
1	5	19
2		12
3	3	7
4		5
5		3
6	1	
8		1

**Table 5:** Numbers At Risk (Months).

0	24
1	24
2	15
3	12
4	10
6	7
7	4
8	3
10	2

## Data Availability

The de-identified raw data supporting the conclusions of this article are available on reasonable request from the corresponding author (ayrton.bangolo@hmhn.org). The data are not publicly available due to strict institutional privacy policies and federal regulations concerning Protected Health Information (PHI) under HIPAA.

## Abbreviations

ASCL1	Achaete-scute homolog 1
ASTCT	American Society for Transplantation and Cellular Therapy
BBB	Blood–brain barrier
BiTE	Bispecific T-cell engager
B2M	Beta-2-microglobulin
CI	Confidence interval
CNS	Central nervous system
CR	Complete response
CRPC-NE	Castration-resistant neuroendocrine prostate cancer
CRS	Cytokine release syndrome
DCR	Disease control rate
DLL3	Delta-like ligand 3
ECOG PS	Eastern Cooperative Oncology Group performance status
EMR	Electronic medical records
EPSCC	Extrapulmonary small cell carcinoma
FDA	Food and Drug Administration
GEJ	Gastroesophageal junction
ICANS	Immune effector cell-associated neurotoxicity syndrome
IL-6	Interleukin-6
iORR	Intracranial objective response rate
IRB	Institutional Review Board
MHC	Major histocompatibility complex
NE	Not evaluable
ORR	Objective response rate
OS	Overall survival
PD	Progressive disease
PDL-1	Programmed death-ligand 1
PFS	Progression-free survival
PR	Partial response
RECIST	Response Evaluation Criteria in Solid Tumors
SCLC	Small cell lung cancer
SD	Stable disease
TME	Tumor microenvironment
